# *N*-(Anthracen-9-ylmeth­yl)adamantan-1-amine

**DOI:** 10.1107/S1600536812022106

**Published:** 2012-05-19

**Authors:** Wei-Qiang Fan, Fu-Xiao Chen

**Affiliations:** a School of Chemistry and Chemical Engineering, Jiangsu University, Zhenjiang 212023, People’s Republic of China; b School of the Environment, Jiangsu University, Zhenjiang 212023, People’s Republic of China

## Abstract

In the crystal stucture of the of the title compound, C_25_H_27_N, stong π–π inter­actions are found between adjacent anthracene fragments, with a shortest centroid–centroid distance of 3.5750 (9) Å.

## Related literature

Anthracene derivatives have been widely used in the field of anion recognition, metal ion fluorescent sensors, as well as pH sensors, see: Bernhardt *et al.* (2001 ▶), Chen & Chen (2004 ▶); Gunnlaugsson *et al.* (2003 ▶); Kim & Yoon (2002 ▶)
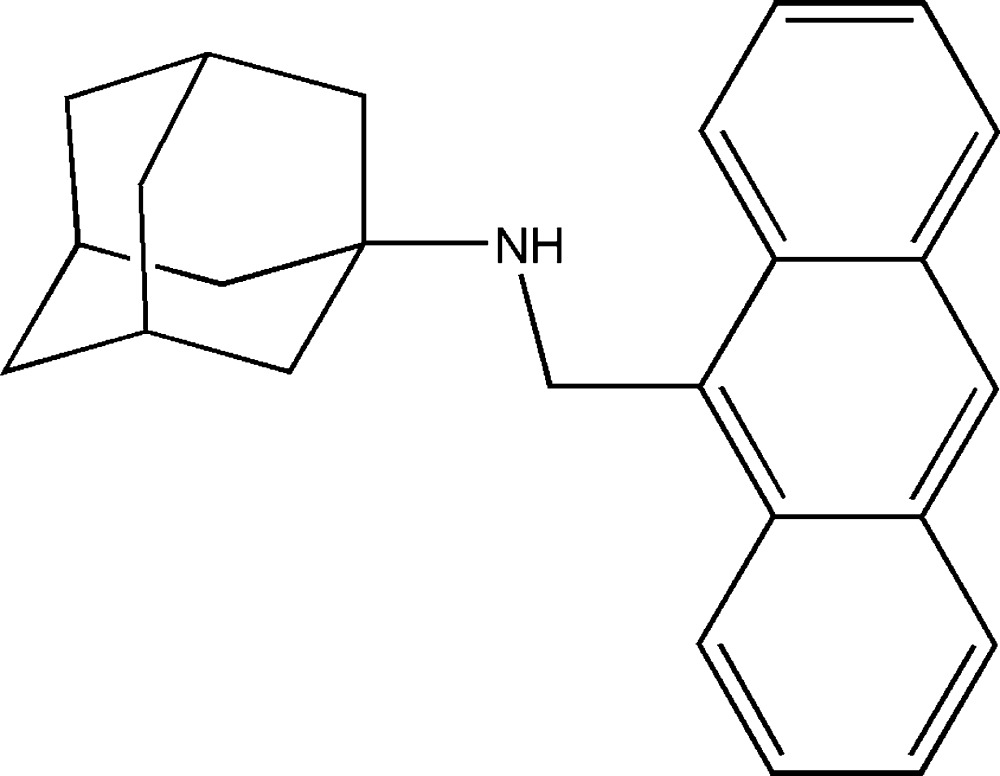


## Experimental

### 

#### Crystal data

C_25_H_27_N
*M* *_r_* = 341.26
Orthorhombic, 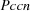

*a* = 9.9546 (4) Å
*b* = 42.1921 (19) Å
*c* = 8.6133 (4) Å
*V* = 3617.6 (3) Å^3^
*Z* = 8
Mo *K*α radiation
μ = 0.07 mm^−1^
*T* = 293 K
0.35 × 0.24 × 0.20 mm

#### Data collection

Bruker APEX CCD area-detector diffractometer
Absorption correction: multi-scan (*SADABS*; Bruker, 1999 ▶) *T*_min_ = 0.975, *T*_max_ = 0.998
18689 measured reflections
3594 independent reflections
3089 reflections with *I* > 2σ(*I*)
*R*_int_ = 0.037

#### Refinement

*R*[*F* ^2^ > 2σ(*F* ^2^)] = 0.039
*wR*(*F* ^2^) = 0.104
*S* = 1.03
3594 reflections
238 parameters
1 restraint
H atoms treated by a mixture of independent and constrained refinement
Δρ_max_ = 0.30 e Å^−3^
Δρ_min_ = −0.20 e Å^−3^

### 

Data collection: *SMART* (Bruker, 1999 ▶); cell refinement: *SAINT* (Bruker, 1999 ▶); data reduction: *SAINT*; program(s) used to solve structure: *SHELXS97* (Sheldrick, 2008 ▶); program(s) used to refine structure: *SHELXL97* (Sheldrick, 2008 ▶); molecular graphics: *SHELXTL-Plus* (Sheldrick, 2008 ▶); software used to prepare material for publication: *SHELXL97*.

## Supplementary Material

Crystal structure: contains datablock(s) I, global. DOI: 10.1107/S1600536812022106/aa2056sup1.cif

Structure factors: contains datablock(s) I. DOI: 10.1107/S1600536812022106/aa2056Isup2.hkl

Supplementary material file. DOI: 10.1107/S1600536812022106/aa2056Isup3.cml

Additional supplementary materials:  crystallographic information; 3D view; checkCIF report

## Figures and Tables

**Fig. 1.**
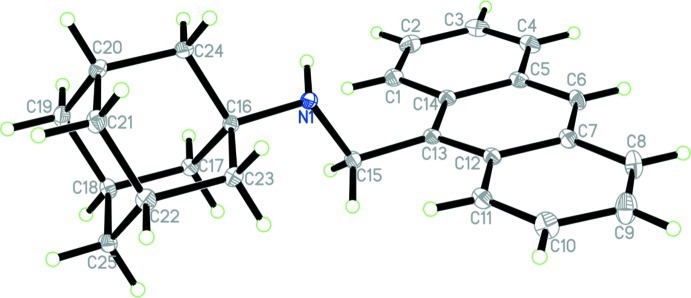
The molecular structure of the title compound. Thermal displacement ellipsoids are drawn at the 30% probability level.

**Fig. 2.**
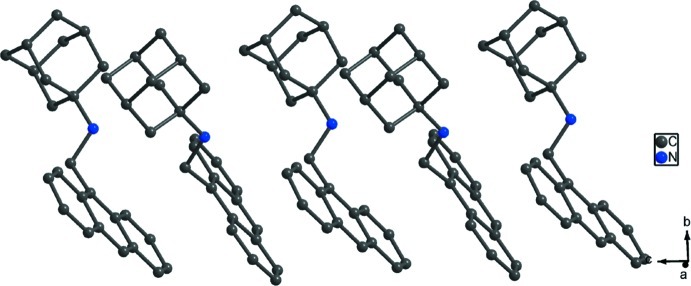
The one-dimensional supramolecular chains linked by π–π interactions. H atoms are omitted for clarity.

## supplementary crystallographic information

### Comment

Anthracene derivatives have been widely used in the field of anion recognition, metal ionfluorescent sensors, as well as pH sensors (Gunnlaugsson *et al.*, 2003; Chen & Chen, 2004; Kim & Yoon, 2002; Bernhardt *et al.* 2001) because of their excellent photophysical properties and high fluorescence.

In the crystals of the title compound (Fig. 1), there are two π–π interactions between benzene rings of the ajacent anthracene fragments with the distances *Cg*1···*Cg*2^i^ = 3.5750 (9) Å and *Cg*1···*Cg*3^i^ = 4.0043 (10) Å. (*Cg*1, *Cg*2 and *Cg*3 are the centroids of the rings [C1/C5 and C14], [C5/C7 and C12/C14] and [C7/C12], respectively; symmetry code: (i) 1/2 - *x*, *y*, *z* + 1/2) forming one-dimensional supramolecular chains along *c* axis direction (Fig. 2).

### Experimental

9-Anthracenecarboxaldehyde (2.06 g, 10 mmol) was added into a solution of amantadine (1.51 g, 10 mmol) in ethanol. Yellow precipitate was formed atfer string for 1 h. The yellow Schiff base was filtrated and dryed. NaBH_4_ (7.56 g, 20 mmol) was added into a solution of the Schiff base in anhydrous methanol (120 ml). After 3 h, the white solid, 9-[(adamantan-1-ylamino)methyl]anthracene, was obtained by reduced pressure distillation, extraction and drying. The colourless block-shaped crystals of the title compound suitable for X-ray analysis were obtained by recrystallization from ethanol.

### Refinement

H atom bonded to N was located in a difference Fourier map and refined isotropically with a bond restraint of N—H= 0.85 Å and *U*_iso_(H) = 1.5 *U*_eq_(N). Other H atoms were placed in calculated positions with C—H distances 0.93 (aromatic), 0.97 Å (methylene) and 0.97 Å (methine) and refined as riding with *U*_iso_(H) = 1.2*U*_eq_(C).

### Crystal data

**Table:**

C_25_H_27_N	*F*(000) = 1472
*M**_r_* = 341.26	*D*_x_ = 1.253 Mg m^−^^3^
Orthorhombic, *P**c**c**n*	Mo *K*α radiation, λ = 0.71073 Å
Hall symbol: -P 2ab 2ac	Cell parameters from 3594 reflections
*a* = 9.9546 (4) Å	θ = 1.0–26.0°
*b* = 42.1921 (19) Å	µ = 0.07 mm^−^^1^
*c* = 8.6133 (4) Å	*T* = 293 K
*V* = 3617.6 (3) Å^3^	Block, colorless
*Z* = 8	0.35 × 0.24 × 0.20 mm

### Data collection

**Table:**

Bruker APEX CCD area-detector diffractometer	3594 independent reflections
Radiation source: fine-focus sealed tube	3089 reflections with *I* > 2σ(*I*)
Graphite monochromator	*R*_int_ = 0.037
ω scans	θ_max_ = 26.0°, θ_min_ = 1.0°
Absorption correction: multi-scan (*SADABS*; Bruker, 1999)	*h* = −8→12
*T*_min_ = 0.975, *T*_max_ = 0.998	*k* = −52→50
18689 measured reflections	*l* = −10→10

### Refinement

**Table:**

Refinement on *F*^2^	Primary atom site location: structure-invariant direct methods
Least-squares matrix: full	Secondary atom site location: difference Fourier map
*R*[*F*^2^ > 2σ(*F*^2^)] = 0.039	Hydrogen site location: inferred from neighbouring sites
*wR*(*F*^2^) = 0.104	H atoms treated by a mixture of independent and constrained refinement
*S* = 1.03	*w* = 1/[σ^2^(*F*_o_^2^) + (0.0431*P*)^2^ + 1.8749*P*] where *P* = (*F*_o_^2^ + 2*F*_c_^2^)/3
3594 reflections	(Δ/σ)_max_ < 0.001
238 parameters	Δρ_max_ = 0.30 e Å^−^^3^
1 restraint	Δρ_min_ = −0.20 e Å^−^^3^

### Special details

**Table:**

Geometry. All s.u.'s (except the s.u. in the dihedral angle between two l.s. planes) are estimated using the full covariance matrix. The cell s.u.'s are taken into account individually in the estimation of s.u.'s in distances, angles and torsion angles; correlations between s.u.'s in cell parameters are only used when they are defined by crystal symmetry. An approximate (isotropic) treatment of cell s.u.'s is used for estimating s.u.'s involving l.s. planes.
Refinement. Refinement of *F*^2^ against ALL reflections. The weighted *R*-factor *wR* and goodness of fit *S* are based on *F*^2^, conventional *R*-factors *R* are based on *F*, with *F* set to zero for negative *F*^2^. The threshold expression of *F*^2^ > σ(*F*^2^) is used only for calculating *R*-factors(gt) *etc*. and is not relevant to the choice of reflections for refinement. *R*-factors based on *F*^2^ are statistically about twice as large as those based on *F*, and *R*- factors based on ALL data will be even larger.

### Fractional atomic coordinates and isotropic or equivalent isotropic displacement parameters (Å^2^)

**Table:**

	*x*	*y*	*z*	*U*_iso_*/*U*_eq_	
C1	0.07687 (14)	0.06244 (3)	0.02106 (16)	0.0213 (3)	
H1	0.0190	0.0763	0.0720	0.026*	
C2	0.02463 (14)	0.03905 (3)	−0.06924 (16)	0.0251 (3)	
H2	−0.0681	0.0372	−0.0795	0.030*	
C3	0.10948 (15)	0.01740 (3)	−0.14794 (16)	0.0263 (3)	
H3	0.0724	0.0015	−0.2089	0.032*	
C4	0.24486 (15)	0.01993 (3)	−0.13433 (15)	0.0239 (3)	
H4	0.2998	0.0057	−0.1866	0.029*	
C5	0.30441 (14)	0.04417 (3)	−0.04106 (15)	0.0189 (3)	
C6	0.44299 (14)	0.04664 (3)	−0.02481 (15)	0.0206 (3)	
H6	0.4980	0.0321	−0.0753	0.025*	
C7	0.50153 (13)	0.07034 (3)	0.06532 (15)	0.0192 (3)	
C8	0.64357 (14)	0.07199 (3)	0.08598 (18)	0.0275 (3)	
H8	0.6984	0.0572	0.0370	0.033*	
C9	0.70002 (15)	0.09479 (3)	0.1760 (2)	0.0320 (4)	
H9	0.7925	0.0951	0.1911	0.038*	
C10	0.61811 (14)	0.11826 (3)	0.24708 (17)	0.0266 (3)	
H10	0.6577	0.1342	0.3061	0.032*	
C11	0.48252 (13)	0.11763 (3)	0.22964 (15)	0.0201 (3)	
H11	0.4309	0.1334	0.2755	0.024*	
C12	0.41699 (13)	0.09318 (3)	0.14216 (14)	0.0165 (3)	
C13	0.27606 (13)	0.09040 (3)	0.13158 (14)	0.0164 (3)	
C14	0.21886 (13)	0.06624 (3)	0.03960 (14)	0.0177 (3)	
C15	0.18568 (13)	0.11250 (3)	0.22289 (14)	0.0173 (3)	
H15A	0.1067	0.1010	0.2570	0.021*	
H15B	0.2330	0.1199	0.3145	0.021*	
C16	0.07339 (12)	0.16531 (3)	0.21643 (14)	0.0148 (3)	
C17	−0.03739 (12)	0.15278 (3)	0.32484 (14)	0.0161 (3)	
H17A	−0.1027	0.1409	0.2647	0.019*	
H17B	0.0018	0.1385	0.4007	0.019*	
C18	−0.10802 (12)	0.18021 (3)	0.40888 (15)	0.0178 (3)	
H18	−0.1777	0.1717	0.4777	0.021*	
C19	−0.17216 (13)	0.20239 (3)	0.28916 (16)	0.0211 (3)	
H19A	−0.2167	0.2198	0.3417	0.025*	
H19B	−0.2389	0.1909	0.2293	0.025*	
C20	−0.06276 (13)	0.21533 (3)	0.18062 (16)	0.0204 (3)	
H20	−0.1037	0.2294	0.1034	0.024*	
C21	0.04073 (13)	0.23400 (3)	0.27656 (16)	0.0214 (3)	
H21A	0.1096	0.2425	0.2087	0.026*	
H21B	−0.0030	0.2516	0.3287	0.026*	
C22	0.10463 (13)	0.21183 (3)	0.39676 (15)	0.0193 (3)	
H22	0.1706	0.2237	0.4581	0.023*	
C23	0.17531 (12)	0.18428 (3)	0.31292 (15)	0.0172 (3)	
H23A	0.2450	0.1926	0.2454	0.021*	
H23B	0.2174	0.1705	0.3887	0.021*	
C24	0.00819 (13)	0.18773 (3)	0.09765 (15)	0.0185 (3)	
H24A	−0.0565	0.1761	0.0357	0.022*	
H24B	0.0766	0.1960	0.0285	0.022*	
C25	−0.00415 (13)	0.19856 (3)	0.50496 (15)	0.0191 (3)	
H25A	0.0364	0.1846	0.5811	0.023*	
H25B	−0.0479	0.2158	0.5598	0.023*	
N1	0.14369 (11)	0.14005 (2)	0.12914 (12)	0.0168 (2)	
H1N	0.0920 (15)	0.1338 (3)	0.0533 (16)	0.025*	

### Atomic displacement parameters (Å^2^)

**Table:**

	*U*^11^	*U*^22^	*U*^33^	*U*^12^	*U*^13^	*U*^23^
C1	0.0250 (7)	0.0178 (6)	0.0210 (7)	0.0004 (5)	0.0014 (5)	0.0045 (5)
C2	0.0253 (7)	0.0248 (7)	0.0253 (7)	−0.0064 (6)	−0.0025 (6)	0.0061 (6)
C3	0.0386 (8)	0.0200 (7)	0.0204 (7)	−0.0101 (6)	−0.0025 (6)	0.0001 (5)
C4	0.0371 (8)	0.0158 (6)	0.0189 (7)	−0.0018 (6)	0.0031 (6)	−0.0011 (5)
C5	0.0282 (7)	0.0130 (6)	0.0155 (6)	−0.0009 (5)	0.0019 (5)	0.0020 (5)
C6	0.0272 (7)	0.0143 (6)	0.0204 (7)	0.0037 (5)	0.0065 (5)	−0.0012 (5)
C7	0.0231 (7)	0.0146 (6)	0.0199 (7)	0.0013 (5)	0.0042 (5)	0.0016 (5)
C8	0.0223 (7)	0.0218 (7)	0.0384 (9)	0.0026 (5)	0.0077 (6)	−0.0037 (6)
C9	0.0195 (7)	0.0279 (7)	0.0488 (10)	−0.0032 (6)	0.0031 (7)	−0.0058 (7)
C10	0.0268 (7)	0.0208 (7)	0.0323 (8)	−0.0067 (5)	0.0014 (6)	−0.0042 (6)
C11	0.0257 (7)	0.0146 (6)	0.0201 (7)	0.0000 (5)	0.0038 (5)	0.0003 (5)
C12	0.0229 (6)	0.0119 (6)	0.0147 (6)	0.0009 (5)	0.0024 (5)	0.0022 (5)
C13	0.0231 (7)	0.0120 (5)	0.0139 (6)	0.0021 (5)	0.0023 (5)	0.0041 (5)
C14	0.0240 (7)	0.0138 (6)	0.0152 (6)	0.0004 (5)	0.0017 (5)	0.0044 (5)
C15	0.0203 (6)	0.0151 (6)	0.0165 (6)	0.0013 (5)	0.0021 (5)	0.0015 (5)
C16	0.0168 (6)	0.0124 (6)	0.0153 (6)	0.0010 (5)	0.0002 (5)	−0.0002 (5)
C17	0.0173 (6)	0.0141 (6)	0.0170 (6)	−0.0018 (5)	0.0009 (5)	−0.0005 (5)
C18	0.0176 (6)	0.0165 (6)	0.0192 (6)	−0.0003 (5)	0.0038 (5)	−0.0013 (5)
C19	0.0179 (6)	0.0209 (6)	0.0246 (7)	0.0044 (5)	−0.0008 (5)	−0.0036 (5)
C20	0.0249 (7)	0.0156 (6)	0.0207 (7)	0.0052 (5)	−0.0020 (5)	0.0027 (5)
C21	0.0259 (7)	0.0129 (6)	0.0254 (7)	0.0006 (5)	0.0025 (6)	0.0008 (5)
C22	0.0206 (6)	0.0146 (6)	0.0226 (7)	−0.0029 (5)	−0.0024 (5)	−0.0028 (5)
C23	0.0158 (6)	0.0153 (6)	0.0206 (7)	−0.0008 (5)	−0.0005 (5)	0.0016 (5)
C24	0.0215 (6)	0.0180 (6)	0.0160 (6)	0.0020 (5)	−0.0006 (5)	0.0012 (5)
C25	0.0256 (7)	0.0145 (6)	0.0171 (6)	0.0019 (5)	−0.0006 (5)	−0.0022 (5)
N1	0.0212 (6)	0.0145 (5)	0.0147 (5)	0.0037 (4)	−0.0003 (4)	0.0002 (4)

### Geometric parameters (Å, º)

**Table:**

C1—C2	1.3598 (19)	C16—N1	1.4800 (15)
C1—C14	1.4314 (19)	C16—C23	1.5364 (16)
C1—H1	0.9300	C16—C24	1.5373 (16)
C2—C3	1.417 (2)	C16—C17	1.5387 (16)
C2—H2	0.9300	C17—C18	1.5355 (16)
C3—C4	1.357 (2)	C17—H17A	0.9700
C3—H3	0.9300	C17—H17B	0.9700
C4—C5	1.4291 (18)	C18—C19	1.5318 (18)
C4—H4	0.9300	C18—C25	1.5339 (17)
C5—C6	1.3905 (19)	C18—H18	0.9800
C5—C14	1.4407 (18)	C19—C20	1.5357 (18)
C6—C7	1.3937 (18)	C19—H19A	0.9700
C6—H6	0.9300	C19—H19B	0.9700
C7—C8	1.4268 (19)	C20—C24	1.5378 (17)
C7—C12	1.4403 (17)	C20—C21	1.5379 (18)
C8—C9	1.357 (2)	C20—H20	0.9800
C8—H8	0.9300	C21—C22	1.5335 (18)
C9—C10	1.422 (2)	C21—H21A	0.9700
C9—H9	0.9300	C21—H21B	0.9700
C10—C11	1.3584 (19)	C22—C25	1.5346 (18)
C10—H10	0.9300	C22—C23	1.5389 (17)
C11—C12	1.4346 (17)	C22—H22	0.9800
C11—H11	0.9300	C23—H23A	0.9700
C12—C13	1.4107 (18)	C23—H23B	0.9700
C13—C14	1.4110 (17)	C24—H24A	0.9700
C13—C15	1.5155 (16)	C24—H24B	0.9700
C15—N1	1.4760 (15)	C25—H25A	0.9700
C15—H15A	0.9700	C25—H25B	0.9700
C15—H15B	0.9700	N1—H1N	0.873 (13)
			
C2—C1—C14	121.52 (13)	C16—C17—H17A	109.5
C2—C1—H1	119.2	C18—C17—H17B	109.5
C14—C1—H1	119.2	C16—C17—H17B	109.5
C1—C2—C3	120.90 (13)	H17A—C17—H17B	108.1
C1—C2—H2	119.6	C19—C18—C25	109.63 (10)
C3—C2—H2	119.5	C19—C18—C17	109.51 (10)
C4—C3—C2	120.00 (12)	C25—C18—C17	109.03 (10)
C4—C3—H3	120.0	C19—C18—H18	109.6
C2—C3—H3	120.0	C25—C18—H18	109.6
C3—C4—C5	121.12 (13)	C17—C18—H18	109.6
C3—C4—H4	119.4	C18—C19—C20	109.35 (10)
C5—C4—H4	119.4	C18—C19—H19A	109.8
C6—C5—C4	121.45 (12)	C20—C19—H19A	109.8
C6—C5—C14	119.30 (12)	C18—C19—H19B	109.8
C4—C5—C14	119.24 (12)	C20—C19—H19B	109.8
C5—C6—C7	121.65 (12)	H19A—C19—H19B	108.3
C5—C6—H6	119.2	C19—C20—C24	109.84 (10)
C7—C6—H6	119.2	C19—C20—C21	109.27 (11)
C6—C7—C8	121.25 (12)	C24—C20—C21	109.26 (10)
C6—C7—C12	119.45 (12)	C19—C20—H20	109.5
C8—C7—C12	119.29 (12)	C24—C20—H20	109.5
C9—C8—C7	121.07 (13)	C21—C20—H20	109.5
C9—C8—H8	119.5	C22—C21—C20	109.15 (10)
C7—C8—H8	119.5	C22—C21—H21A	109.8
C8—C9—C10	120.16 (13)	C20—C21—H21A	109.8
C8—C9—H9	119.9	C22—C21—H21B	109.8
C10—C9—H9	119.9	C20—C21—H21B	109.8
C11—C10—C9	120.58 (13)	H21A—C21—H21B	108.3
C11—C10—H10	119.7	C21—C22—C25	109.87 (11)
C9—C10—H10	119.7	C21—C22—C23	109.49 (10)
C10—C11—C12	121.59 (12)	C25—C22—C23	109.38 (10)
C10—C11—H11	119.2	C21—C22—H22	109.4
C12—C11—H11	119.2	C25—C22—H22	109.4
C13—C12—C11	123.08 (11)	C23—C22—H22	109.4
C13—C12—C7	119.73 (11)	C16—C23—C22	110.21 (10)
C11—C12—C7	117.18 (12)	C16—C23—H23A	109.6
C12—C13—C14	119.83 (11)	C22—C23—H23A	109.6
C12—C13—C15	120.39 (11)	C16—C23—H23B	109.6
C14—C13—C15	119.75 (11)	C22—C23—H23B	109.6
C13—C14—C1	122.83 (12)	H23A—C23—H23B	108.1
C13—C14—C5	119.96 (12)	C16—C24—C20	110.53 (10)
C1—C14—C5	117.21 (12)	C16—C24—H24A	109.5
N1—C15—C13	111.64 (10)	C20—C24—H24A	109.5
N1—C15—H15A	109.3	C16—C24—H24B	109.5
C13—C15—H15A	109.3	C20—C24—H24B	109.5
N1—C15—H15B	109.3	H24A—C24—H24B	108.1
C13—C15—H15B	109.3	C18—C25—C22	109.40 (10)
H15A—C15—H15B	108.0	C18—C25—H25A	109.8
N1—C16—C23	109.73 (10)	C22—C25—H25A	109.8
N1—C16—C24	107.74 (10)	C18—C25—H25B	109.8
C23—C16—C24	108.55 (10)	C22—C25—H25B	109.8
N1—C16—C17	113.57 (9)	H25A—C25—H25B	108.2
C23—C16—C17	108.90 (10)	C15—N1—C16	115.03 (9)
C24—C16—C17	108.22 (10)	C15—N1—H1N	109.8 (10)
C18—C17—C16	110.81 (10)	C16—N1—H1N	108.6 (10)
C18—C17—H17A	109.5		
			
C14—C1—C2—C3	0.3 (2)	C12—C13—C15—N1	94.66 (13)
C1—C2—C3—C4	−0.3 (2)	C14—C13—C15—N1	−87.49 (13)
C2—C3—C4—C5	0.2 (2)	N1—C16—C17—C18	−178.78 (10)
C3—C4—C5—C6	179.09 (13)	C23—C16—C17—C18	58.63 (13)
C3—C4—C5—C14	−0.04 (19)	C24—C16—C17—C18	−59.20 (13)
C4—C5—C6—C7	179.63 (12)	C16—C17—C18—C19	60.18 (13)
C14—C5—C6—C7	−1.24 (19)	C16—C17—C18—C25	−59.77 (13)
C5—C6—C7—C8	177.65 (13)	C25—C18—C19—C20	60.30 (13)
C5—C6—C7—C12	−0.85 (19)	C17—C18—C19—C20	−59.28 (13)
C6—C7—C8—C9	−178.87 (14)	C18—C19—C20—C24	59.26 (13)
C12—C7—C8—C9	−0.4 (2)	C18—C19—C20—C21	−60.59 (13)
C7—C8—C9—C10	−2.3 (2)	C19—C20—C21—C22	60.28 (13)
C8—C9—C10—C11	1.9 (2)	C24—C20—C21—C22	−59.93 (13)
C9—C10—C11—C12	1.2 (2)	C20—C21—C22—C25	−59.94 (13)
C10—C11—C12—C13	174.98 (13)	C20—C21—C22—C23	60.21 (13)
C10—C11—C12—C7	−3.74 (19)	N1—C16—C23—C22	176.69 (10)
C6—C7—C12—C13	3.06 (18)	C24—C16—C23—C22	59.19 (13)
C8—C7—C12—C13	−175.46 (12)	C17—C16—C23—C22	−58.43 (12)
C6—C7—C12—C11	−178.18 (11)	C21—C22—C23—C16	−60.42 (13)
C8—C7—C12—C11	3.30 (18)	C25—C22—C23—C16	60.03 (13)
C11—C12—C13—C14	178.18 (11)	N1—C16—C24—C20	−178.02 (10)
C7—C12—C13—C14	−3.14 (17)	C23—C16—C24—C20	−59.25 (13)
C11—C12—C13—C15	−3.97 (18)	C17—C16—C24—C20	58.80 (13)
C7—C12—C13—C15	174.71 (11)	C19—C20—C24—C16	−59.76 (13)
C12—C13—C14—C1	−178.69 (11)	C21—C20—C24—C16	60.10 (13)
C15—C13—C14—C1	3.44 (17)	C19—C18—C25—C22	−59.63 (13)
C12—C13—C14—C5	1.06 (17)	C17—C18—C25—C22	60.24 (13)
C15—C13—C14—C5	−176.81 (11)	C21—C22—C25—C18	59.59 (13)
C2—C1—C14—C13	179.54 (12)	C23—C22—C25—C18	−60.63 (13)
C2—C1—C14—C5	−0.22 (18)	C13—C15—N1—C16	−170.68 (10)
C6—C5—C14—C13	1.15 (18)	C23—C16—N1—C15	75.36 (12)
C4—C5—C14—C13	−179.70 (11)	C24—C16—N1—C15	−166.62 (10)
C6—C5—C14—C1	−179.09 (11)	C17—C16—N1—C15	−46.77 (14)
C4—C5—C14—C1	0.06 (17)		
